# Effect of the Application Time of Accentuated Cut Edges (ACE) on Marquette Wine Phenolic Compounds

**DOI:** 10.3390/molecules27020542

**Published:** 2022-01-15

**Authors:** Yiliang Cheng, Jennifer Rae Savits, Aude Annie Watrelot

**Affiliations:** 1 Department of Food Science and Human Nutrition, Iowa State University, 536 Farm House Lane, Ames, IA 50011, USA; ycheng8@iastate.edu; 2 Midwest Grape and Wine Industry Institute, Iowa State University, 536 Farm House Lane, Ames, IA 50011, USA; jsavits@iastate.edu

**Keywords:** anthocyanins, color stability, Marquette, skin fragmentation, tannins, wine aging

## Abstract

Cold-hardy interspecific hybrid grape varieties (*Vitis* spp.) have distinctive chemical compositions such as high acidity, a high content of anthocyanin diglucoside and a low condensed tannins content, compared to *Vitis vinifera* varieties. Considering the importance of phenolic compounds on the quality of red wine, a mechanical maceration technique, accentuated cut edges (ACE), has been evaluated when applied directly to crushed grapes (ACE-C), and 24 h before pressing (ACE-P), to improve the extraction of phenolic compounds. Samples were collected at crushing, bottling, and after five months of aging. Phenolic compounds and color characteristics of the wines were analyzed by high-performance liquid chromatography (HPLC) with diode array and fluorescence detectors and UV-Visible spectrophotometry. The color intensity, non-anthocyanin monomeric compounds and total iron-reactive phenolics content increased after applying ACE, compared to the control (CTL) after aging, and was significantly higher (37%) after ACE-C, compared to ACE-P. However, the concentration of condensed tannins was below the limit of detection in all the samples, indicating that ACE did not help their extraction or further interactions occurred with disrupted cell wall material. Applying ACE at crushing was considered as the optimum time to achieve a higher color stability in Marquette red wines.

## 1. Introduction

Phenolic compounds are responsible for the color, taste, mouthfeel attributes, and some antioxidant properties of wine. Red wine typically contains a higher concentration of phenolic compounds (1800 mg/L gallic acid equivalents (GAE)) than white wine (200 mg/L GAE) [1,2]. Based on the chemical structure, phenolic compounds are divided into two major classes as non-flavonoids and flavonoids. These compounds are mainly found in the grape skin and seed with a relatively higher content than in the grape flesh. During the winemaking process, they are extracted from crushed grapes and are released into wines during alcoholic fermentation and maceration. Non-flavonoid phenolic compounds include hydroxycinnamic acids (e.g., caftaric, coutaric acids), hydroxybenzoic acids (e.g., gallic acid), and stilbenes (e.g., resveratrol). Flavonoids are the central group of phenolic compounds, accounting for 74% of the total monomeric phenolic compounds in red wine, such as anthocyanin, flavonols, and flavanols [3].

Anthocyanins are the most abundant monomeric phenolic compounds, which are mainly extracted from the grape skins during the red winemaking process and are responsible for the red color. In *Vitis vinifera* grapes, anthocyanins are mainly mono-glucosides, while in native American and interspecific hybrid grapes, anthocyanin di-glucoside are also present [4]. The stabilization of monomeric anthocyanins is an essential step to maintaining the color of red wine during wine production and aging [1,5]. Condensed tannins are oligomers and polymers of flavanols, which are mainly located in grape seeds and skins [6]. In addition to forming stable compounds with anthocyanins, condensed tannins are responsible for wine astringency, mouthfeel, and contribute to biological and overall wine quality. Along with the chemical structure and tissue distribution differences, skin-derived condensed tannins are usually perceived as “velvety” and extracted prior to seed-derived tannins [7].

Unlike *V. vinifera* grape varieties, native American grapes and interspecific hybrid species (e.g., *Vitis rupestris*, *Vitis riparia*, and *Vitis labrusca*) are primarily grown in the U.S. Midwest, due to their resistance to harsh cold winters and to diseases [8]. One of the most popular interspecific grape hybrid species is Marquette, a cross between MN 1094 and Ravat 262. Its lineage includes *Vitis riparia*, *Vitis vinifera,* such as Pinot noir variety, and other *Vitis* species. Marquette has a relatively high resistance to downy mildew, high vigor, and can tolerate temperatures down to −34 °C [8]. Wines produced from these cold-hardy interspecific grapes have a high anthocyanin diglucoside content representing ~81% and ~67% of total anthocyanins content in Corot noir and in Marquette wines, respectively [9]. In addition, those wines are poor in tannins, leading to some challenges for wine color stability and mouthfeel. According to Springer and Sacks [10], interspecific hybrid grapes and wines usually contained 1.8-fold and 5.5-fold lower tannins than *V. vinifera* grapes and wines, respectively. The same authors mentioned that tannin extractability percentage was 2.2 to 5.7% in interspecific hybrid varieties, which was lower than the percentage of extractability of 8 to 22% in *V. vinifera* varieties. Therefore, this requires a better understanding of tannin reactivity, interaction, and retention mechanisms in interspecific hybrid grapes and wines.

An innovative vinification technique, accentuated cut edges (ACE), was previously described and showed an enhancement in Pinot noir [11,12] and Shiraz [13] wine color stability and condensed tannin content after the mechanical breakdown of grape skins. ACE treatment increased the concentration of condensed tannin by 3 to 7-fold in Pinot noir aged wine [11,12] because of the fragmentation and reduction in size of grape skins, promoting the release of phenolic compounds. In addition to the enhancement of phenolics released by ACE, the formation of stable anthocyanin-based pigments was also improved and these wines contained 50 to 70% higher color density after aging in the same studies [11,12]. Although the intensified breakdown of grape skin also significantly increased the concentration of tannins when applied to Shiraz wine, less effect on color attributes was observed compared to the studies on Pinot noir [13]. These inconsistent results showed that the effects of ACE on the extraction of phenolic compounds remain unknown and could be beneficial to wines with low tannin content.

Limited studies focused on the effect of winemaking techniques on the extraction of phenolic compounds from cold-hardy interspecific hybrid grapes. Therefore, the Marquette grape variety was selected to conduct the study on the effect of ACE treatment on wine phenolics extraction and color characteristics. Moreover, with the differences in solubility, anthocyanin and condensed tannins reach their maximum extractability at different stages during alcoholic fermentation [14]. For this reason, the application time of ACE might also play an important role in the efficiency of grape phenolic extraction. The objective of this study was to evaluate the effect of the ACE technique and its application time, at crushing and 24 h before pressing, on phenolic extraction and color stability of the interspecific hybrid red wine, cv. Marquette. It was hypothesized that ACE technique would disrupt skin cell wall material and improve the extraction of tannins and other phenolic compounds of Marquette grapes, in order to help stabilize wine color over time, especially when applied during alcoholic fermentation.

## 2. Results

### 2.1. Basic Chemical Properties

The basic chemical properties of the musts and the wines are shown in Table 1. At crushing, ACE did not significantly affect the pH, TA, and sugar content of the musts. The malic acid was 2-fold higher than tartaric acid in the musts. The musts had an average °Brix of 25, a pH of 3.3, and TA of approximately 12 g/L. At bottling, ACE-C treated wines contained the highest TA, ethanol (%, *v*/*v*), and tartaric acid content. No significant difference was observed for those chemical parameters between ACE-P and the control. After five months of aging, the ACE-C treated wines showed the lowest pH (3.79) and highest TA (8.23 g/L). The highest ethanol concentration was found in ACE-C wine (14.57%) and the lowest in ACE-P wine (13.52%). ACE-P showed the lowest content of tartaric acid and lactic acid.

### 2.2. Color Intensity and Hue

**Figure 1:**
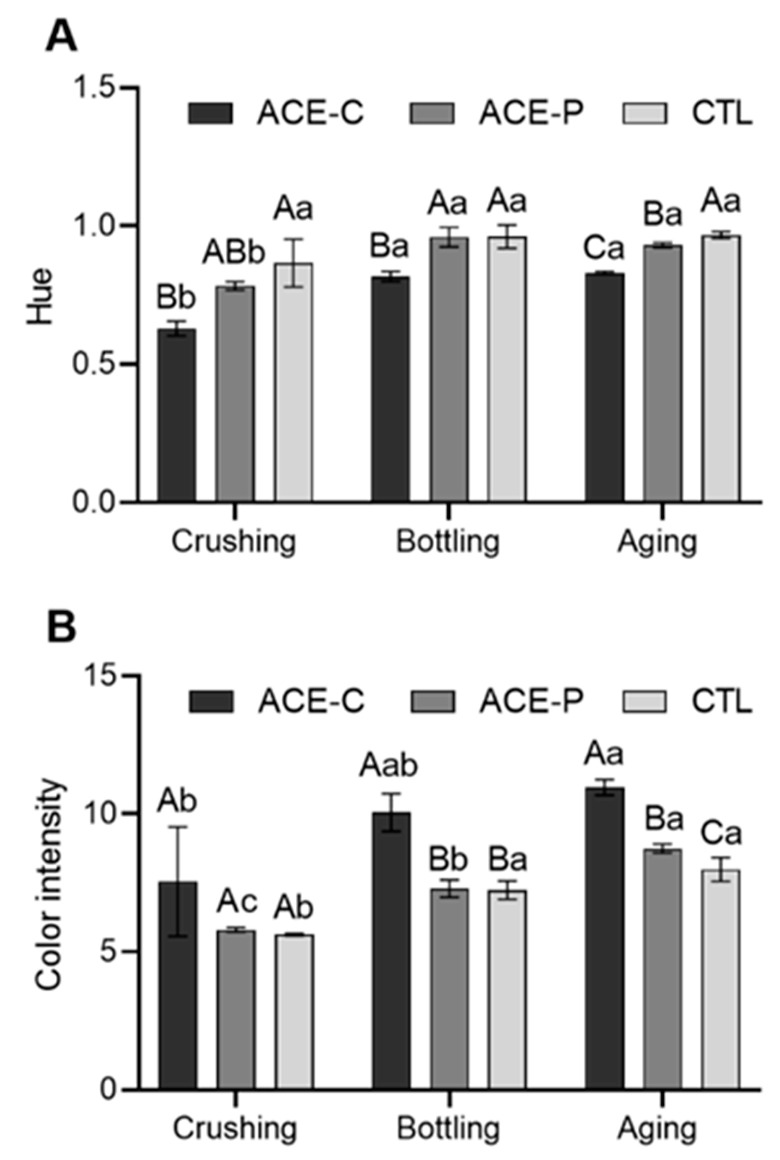
Color characteristics in Marquette grape musts and red wines: (**A**) Hue and (**B**) Color intensity. The error bars represent standard deviation of the mean (*n* = 2). Uppercase letters indicate significant difference (*p* < 0.05) among treatments within same time point. Lowercase letters indicate significant difference (*p* < 0.05) within same sample over time.

The ACE treatment led to a decrease in hue and an increase in color intensity throughout the process (Figure 1). At crushing, ACE-C treated must showed a significantly lower (28%) hue than the control. The ACE-C wine exhibited a significantly lower hue at bottling (15%) and after aging (14%) than control wine, whereas ACE-P treatment only caused a significantly lower hue by 4% after aging. The hue of the control was stable without significant difference throughout the process, whereas the hue of ACE-C and ACE-P significantly increased from crushing to bottling by 30% and 23%, respectively. ACE-C treatment had no significant effect on the color intensity at crushing, but it resulted in the color intensity of wine being significantly higher than the control by 39% at bottling and 37% after aging. After aging, the highest color intensity was observed in ACE-C wines, followed by ACE-P, significantly higher than the control.

### 2.3. Monomeric Phenolic Compounds

**Figure 2:**
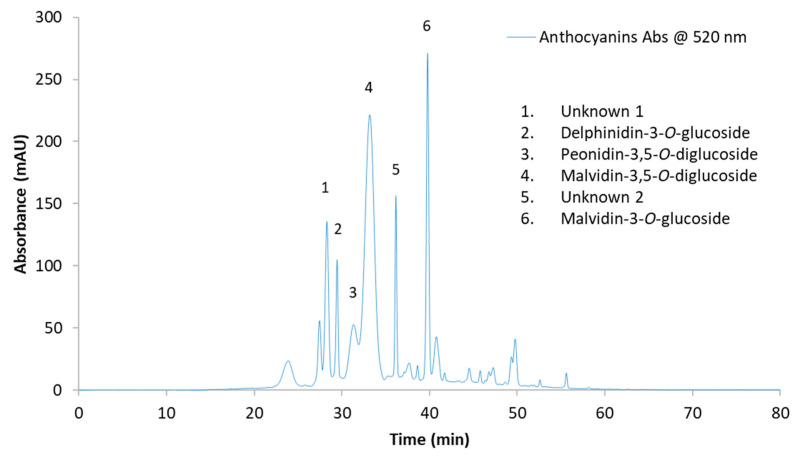
HPLC-DAD chromatogram at 520 nm of anthocyanins from CTL sample after aging. From left to right: unknown 1 (RT 28.287 min); delphinidin-3-*O*-glucoside (RT 29.448 min); peonidin-3,5-*O*-diglucoside (RT 31.330 min); malvidin-3,5-*O*-diglucoside (RT 33.175 min); unknown 2 (RT 36.153 min); malvidin-3-*O*-glucoside (RT 39.772).

Six monomeric anthocyanins were detected and quantified in the samples, of which two anthocyanins are unknown (Figure 2), with malvidin-3,5-*O*-diglucoside (M35DG) as the most abundant anthocyanin in all samples throughout the process (Table 2). At crushing, only three individual anthocyanins (peonidin-3,5-*O*-diglucoside (P35DG), M35DG, and malvidin-3-*O*-glucoside (M3G)) were detected. The concentration of M3G was lower than the limitation of quantification (12.71 mg/L) in the control. No significant difference in total anthocyanin content was observed between the treatments at crushing. At bottling, no significant difference in total anthocyanin content was observed among the three treatments, but a significant increase in the total content within the same treatment was observed from crushing to bottling. The control had the highest increase from crushing to bottling, i.e., 4.4-fold, followed by ACE-P (3.5-fold) and ACE-C wines (2.5-fold). Specifically, ACE-P treated wine showed a comparable concentration of individual anthocyanins to the control, except malvidin-3-*O*-glucoside (45 mg/L), which was significantly lower than the control (54 mg/L). After aging, only ACE-P wines had significantly lower total anthocyanins (241 mg/L) than the control wines (273 mg/L). During aging, the decrease in total anthocyanins in the aged wine ranged from 22% to 25%, depending on the treatment. In ACE-C and ACE-P aged wines, anthocyanin unknown 1, P35DG, and M35DG contents were lower than (*p* < 0.05) in control aged wines. ACE-C treatment had no significant effect on the content of M3G and total anthocyanins in aged wine. In comparison, ACE-P treatment resulted in aged wine with significantly lower concentrations of unknown 1 (4%), unknown 2 (31%), M3G (20%), and total anthocyanins (13%) when compared to ACE-C treatment.

The non-anthocyanin monomeric phenolic compounds quantified in the musts and wines are shown in Table 3. At crushing, the total content of non-anthocyanin monomeric phenolics was the highest in ACE-C must (43 mg/L), followed by ACE-P (28 mg/L) and the control (17 mg/L) without significant difference among treatments. ACE-C must also showed the highest concentration of (+)-catechin, (-)-epicatechin, caftaric acid, and quercetin-3-*O*-glucoside, although no significant difference was found in these concentrations among the treatments. In comparison, (+)-catechin was the primary non-anthocyanin phenolic compound in all musts, accounting for ~47% of the total content. At bottling, ACE-C and ACE-P wines contained a significantly higher concentration of total non-anthocyanin phenolic compounds (an increase of 43% and 6%, respectively) than the control wine. Gallic acid and (+)-catechin were 1.3 and 1.6-fold greater in ACE-C than in the control wines. Although ACE-P treatment resulted in significant increases in most of the individual compounds compared to the control, the significant reduction in quercetin-3-*O*-glucoside (14%) and myricetin (14%) was found in ACE-P wines. After aging, the non-anthocyanin phenolic compounds concentration in ACE-C wines was significantly higher than the ACE-P and the control wines. ACE-C resulted in a 40% increase in the total non-anthocyanin phenolics content, while ACE-P resulted in a 4% increase in aged wines, compared with the control. Regarding the effect of the winemaking process time, gallic acid and myricetin contents from all wines showed significant increases, but the quercetin-3-*O*-glucoside content was significantly lower than the ACE-P and the control after aging. The rest of the non-anthocyanin phenolics content remained stable over time.

### 2.4. Iron-Reactive Phenolic Compounds and Tannins Content

**Figure 3:**
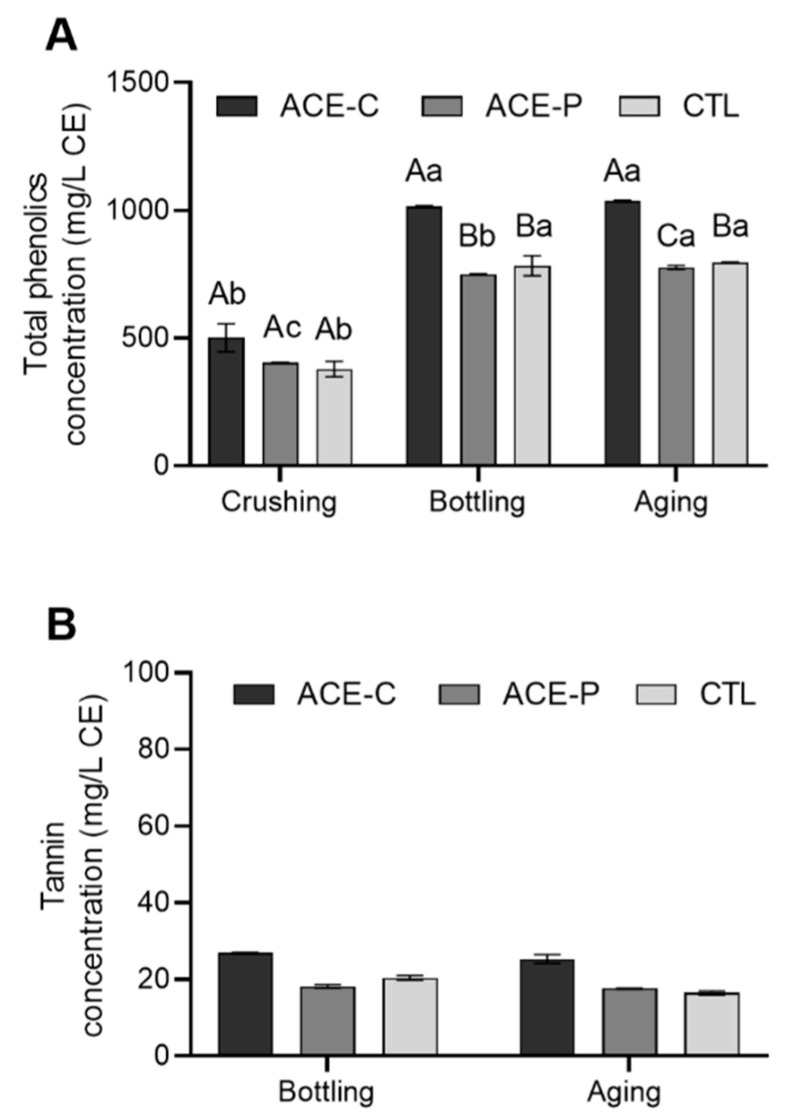
(**A**) Iron-reactive phenolic compounds content and (**B**) Tannin content in Marquette grape musts and red wines. The error bars represent standard derivation of the mean (*n* = 2). Uppercase letters indicate significant difference (*p* < 0.05) among treatments within same time point. Lowercase letters indicate significant difference (*p* < 0.05) within same sample over time.

In all samples, the concentration of total iron-reactive phenolic compounds significantly increased from crushing until bottling but remained stable during aging (Figure 3A). ACE-C musts had the highest average total phenolic contents (502 mg/L). The total phenolic compounds of all wines increased by an average of 90% from the must to the bottled wine. At bottling, no significant difference in total phenolic contents between ACE-P and the control was found, whereas ACE-C wines contained a significantly higher total phenolic compound content than control wines. After aging, the total phenolics contents in all wines ranged from 777 to 1037 mg/L. ACE-C wine had the highest content, followed by the control and ACE-P wine. Compared to the control, ACE-C treatment significantly increased the total phenolics content by 30%, while ACE-P treatment significantly decreased this content by 3%. The tannin concentration of all wines was measured by protein-precipitation assay (Figure 3B). However, the tannin contents from all finished wines were below the limit of quantification (LOQ), which was 140 mg/L ((+)-catechin equivalent), according to Jensen et al. [15].

## 3. Discussion

After alcoholic and malolactic fermentation, the levels of alcohol and acidity were higher in ACE-C wines at bottling and after aging. These differences between ACE-C and the control suggest that ACE-C treatment may promote the release of organic acids and sugars to affect the final physicochemical properties in Marquette wines when applying this skin fragmentation technique at crushing. Conversely, ACE-P did not affect the acidity and showed significantly lower concentrations of alcohol and tartaric acids, which may be because it has been applied at the endpoint of fermentation.

The extraction of phenolic compounds plays a notable role in determining wine quality attributes. One of the most important characteristics is color, which is associated with the composition of monomeric anthocyanins. The anthocyanin composition in Marquette wine was in agreement with previous work where the total monomeric anthocyanin concentration was 255 mg/L, with M35DG accounting for 153 mg/L, M3G accounting for 29 mg/L, and P35DG accounting for 18 mg/L [9]. The application of ACE significantly improved Marquette wine color intensity without a negative impact on total anthocyanin content, which is in agreement with previous works on *V. vinifera* cv. Shiraz and Pinot noir wines [11,13]. Especially, ACE-C wine showed higher color intensity than ACE-P wine, which was similar to previous findings in Pinot noir wine. According to Sparrow et al. [12], the color intensity of Pinot noir wine color increased by 52% when ACE maceration was applied on the first day of fermentation, whereas only an increase of 29% was achieved when applied on day 5 of a six-day fermentation period.

As a water-soluble phenolic compound mainly located in the grape skin cells, anthocyanin is released, and ACE-C treatment strongly promoted diffusion through the mechanically broken skin fragments [12,16,17]. The timing of ACE-P treatment was near the endpoint of fermentation, after the anthocyanin extraction had reached a plateau. Thus, the effect of ACE on anthocyanin extraction was weakened when applying ACE later in the alcoholic fermentation and maceration step. Concerning the hue, ACE-C treated wine resulted in a significantly lower hue than the ACE-P wines and the control. This finding was not in agreement with other studies that showed no impact of the ACE treatment on the hue of Shiraz [13] and Pinot noir wines [12]. However, the anthocyanin and other phenolic compounds content in Marquette wines vs. Shiraz and Pinot noir wines are very different in concentration and structure. For instance, more than 70% of monomeric anthocyanins in Marquette wine at bottling (Table 2) are anthocyanin diglucoside form, which showed different color or hue angles and reaction rates to form stable pigments [4]. This might explain the differences observed.

Red wine color and hue are associated with the type and content of phenolic compounds and the wine pH at bottling. After aging, ACE-C wine showed significantly lower concentration in the anthocyanin unknown 1, P35DG, and M35DG when compared to the control. In addition, the pH of ACE-C wines was lower than in the other wines, which might explain the differences in hue and color intensity between ACE-C wines and ACE-P and control wines. The application of ACE at crushing led to the disruption of grape skins into small fragments, which increased the diffusion and extraction rates of those anthocyanins during the first days of fermentation.

Most non-anthocyanin monomeric phenolics contents remained stable during aging, while the content of gallic acid significantly increased. The total content of non-anthocyanin monomeric phenolics in control wines at bottling (187 mg/L) was higher than previously observed in Marquette wines (83 mg/L) [9]. The ACE-C treatment resulted in a 1.4-fold increase in non-anthocyanin monomeric phenolic compounds, while ACE-P resulted in a 1-fold increase. All the detected monomeric phenolic compounds were in higher content (*p* < 0.05) in ACE-C than ACE-P and control at bottling and after aging. This suggests that some monomeric compounds were extracted from small fragments of grape skins and flesh. In addition, the application of ACE-C on musts facilitated the extraction of phenolics, compared to the application of ACE 24 h prior to pressing. Although there was a lack of reported data on the effect of ACE treatment on non-anthocyanin monomeric phenolic compound extraction in wines, an impact of ACE treatment applied at crushing on total phenolic compound concentration was observed and was consistent with previous work on Shiraz [13].

The overall reduction in monomeric anthocyanin content and the increase in color intensity during aging may be due to a sequence of chemical reactions between anthocyanins themselves and other compounds (e.g., quercetin and condensed tannins), such as self-association, co-pigmentation, hydrolysis, oxidation, condensation, or adsorption to other compounds [18,19]. ACE applied at crushing (ACE-C) did not have any effect on the total anthocyanin content during aging. However, ACE applied prior to pressing (ACE-P) showed a decrease in total anthocyanin content and increased color intensity. This suggested that ACE applied at crushing only impacted the rate of anthocyanin extraction and stability of the color, while ACE applied prior to pressing likely led to the formation of new pigments. The low condensed tannin concentration in the wines suggested that polymeric pigments were not formed in all samples.

As previously observed, the tannin content in interspecific hybrid wines tend to be much lower than in *V. vinifera* wines, suggesting that condensed tannins are retained from cell wall material in interspecific hybrid grapes throughout the winemaking process [10]. In this study, the content of tannins in the wines was below the limit of quantification of the method used. This is in agreement with the study of Springer and Sacks [10] when using the protein precipitation method to quantify the tannin contents in wine made from interspecific hybrid grapes. The use of ACE to improve the tannin concentration by 3-fold in Marquette wines was not obtained, which was in contradiction with previous study on ACE on Pinot noir wines [12]. It is well known that tannins can strongly interact with grape cell wall material such as protein and pectin during grape processing [20,21,22]. ACE treatment could mechanically break skins into smaller fragments and provide more available contact surfaces to extract water-soluble phenolics such as anthocyanins. However, the extractability of condensed tannin within interspecific hybrid cold-hardy grapes was difficult to improve due to the potential interactions between cell wall material and tannins. According to Nicolle et al. [23], the presence of pomace or cell wall material still hindered the improvement of tannin retention in wine made by cold-hardy Frontenac grapes, even when bentonite protein-finning agent and enological tannin addition are involved. These results indicate the necessity of elucidating the molecular interactions between condensed tannins and other compounds such as anthocyanin di-glucoside, polysaccharides, and proteins. Further work will be carried out to investigate the impact of ACE on the cell wall structure, protein, and polysaccharide content to improve the extraction of tannins from cold-hardy interspecific hybrid grapes.

## 4. Materials and Methods

### 4.1. Chemicals and Standards

Hydrochloric acid, sodium hydroxide, glacial acetic acid, ferric chloride hexahydrate, *ortho*-phosphoric acid (≥85%), acetonitrile, sulfuric acid, L-(+)-tartaric acid, D-fructose, and 0.1 N sodium hydroxide were purchased from Fisher Scientific (Santa Clara, CA, USA). Sodium chloride, bovine serum albumin (BSA), (-)-epicatechin (≥90%), (+)-catechin (≥98%), caffeic acid (≥98%), malic acid (≥99%), and quercetin-3-glucoside (≥98%) were purchased from Sigma-Aldrich (St. Louis, MO, USA). Potassium metabisulfite (≥97%), sodium dodecyl sulfate, and ammonium dihydrogen phosphate (>98%) were purchased from Acros Organics (Geel, Belgium). Triethanolamine was purchased from Aqua Solutions, Inc. (Deer Park, TX, USA). Commercial malvidin-3-*O*-glucoside chloride (oenin) (≥95%) and malvidin-3,5-*O*-diglucoside chloride (malvin) (≥95%) were provided by Extrasynthese (Genay, France). Milli-Q water used for the preparation of solutions was obtained from a Barnstead MicroPure Water Purification System (Thermo scientific^®^, Waltham, MA, USA).

### 4.2. Winemaking Protocol

**Figure 4:**
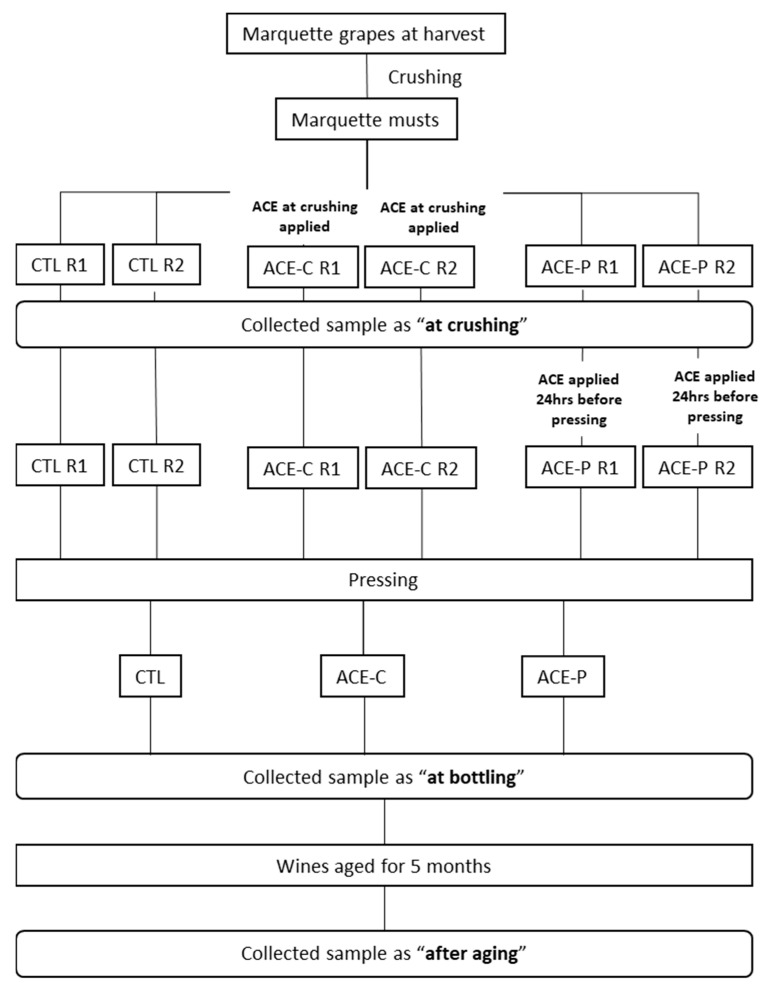
Flowchart of the winemaking protocols with three different treatments: control (CTL), ACE treatment directly after crushing (ACE-C), and ACE treatment 24 h prior to pressing (ACE-P) applied to the Marquette musts. Samples were collected at three time points: crushing, bottling, and after five months aging.

*Vitis* spp. variety Marquette (MN 1094 × Ravat 262) grapes from Brickyard Hill vineyard (Marengo, IA, USA) were mechanically harvested on 28 August 2020 (25.35 ± 0.07 °Brix, pH 3.31 ± 0.01, 11.53 ± 1.46 g/L titratable acidity) and transported to Fireside winery (Marengo, IA, USA). Grapes were processed through the de-stemmer/crusher and then divided into six MacroBins of half-ton each. An immersion blender (MP 350 VV, Robot Coupe®, Ridgeland, MS, USA) was used as the ACE treatment apparatus and applied either at crushing or 24 h prior to pressing with a punch-down movement for 10 min. As shown in Figure 4, two of the MacroBins received ACE treatment directly after crushing and were identified as ACE-C. Two MacroBins received the ACE treatment 24 h prior to pressing and were identified as ACE-P. The remaining two MacroBins contained crushed berries that did not receive any ACE treatment and were identified as the control (CTL). The must of the six MacroBins was inoculated with Lalvin 71B yeast (Lallemand, Petaluma, CA, USA). The cap was punched down twice a day during fermentation for six days. Beta Co-Inoc^TM^ bacteria (Lallemand, Petaluma, CA, USA) was inoculated 48 h after yeast inoculation. After completion of malolactic fermentation (MLF), wines were pressed, and duplicated bins were combined. Potassium metabisulfite was added into the wine at 0.5 mg/L molecular SO_2_. Wines were stored prior to racking-off of lees one month later. The wines were bottled (375 mL) and stored in a cellar with controlled temperature (~12 °C) for five months. Samples were collected at three time points: crushing, bottling, and after five months aging, and stored at −20 °C until required for analysis.

### 4.3. Must and Wine Chemical Properties

The must after crushing was analyzed for total soluble solids (TSS, in Brix) using a digital refractometer Atago^®^ model PAL-1 (Tokyo, Japan). Titratable acidity (TA) and pH of musts and wines were measured, in duplicate or only once, using a digital pH meter ThermoScientific^®^ model Orion Star A211 (Waltham, MA, USA). Titrations were performed with 0.1 N sodium hydroxide to an endpoint of pH 8.2. TA was expressed as g/L tartaric acid equivalents.

All the samples were centrifuged (AccuSpin^TM^ Micro 17 Centrifuge, Thermo Fisher Scientific, Waltham, MA, USA) at 16,200× *g* for 5 min. Organic acids, alcohols, and sugars were quantified in the supernatants using high-performance liquid chromatography (HPLC) (1200 series, Agilent Technologies, Santa Clara, CA, USA) with a diode array detector (DAD) and refractive index detector (RID) (Agilent 1200 series). A Bio-Rad Aminex HPX-87H (Bio-Rad, Hercules, CA, USA) and Bio-Rad fermentation monitoring columns (Bio-Rad, Hercules, CA, USA) with H^+^ guard cartridge were used, and 10 µL of the sample was injected. The mobile phase was 5 mM sulfuric acid with a flow rate of 0.65 mL/min for 35 min. The detection of malic acid was carried out at 210 nm with the DAD. The detection of other organic acids, residual sugars and alcohols was carried out with the RID (cell temperature of 55 °C). Calibration curves for each compound were established with corresponding commercial standards from Bio-Rad (Table S1, Supplementary data). The chemical profile of the samples at crushing, at bottling, and after aging were analyzed in duplicate.

### 4.4. Color Analysis

The hue and color intensity of must and wine samples were determined using a UV-Visible spectrophotometer (Genesys 150, ThermoScientific, Waltham, MA, USA) with a 1 mm path length Quartz cuvettes (Azzota Scientific, Claymont, DE, USA). All the samples were centrifuged at 16,200× *g* for 5 min prior to analysis of the supernatant. The color intensity was calculated as the sum of absorbance values at 420 nm, 520 nm, and 620 nm times the dilution factor 10. The hue was calculated as the ratio of absorbance value at 420 nm to at 520 nm. The color measurement was carried out in duplicate.

### 4.5. Monomeric Phenolic Compound Analysis

Monomeric phenolic compounds of must and wine samples were analyzed using a 1260 Infinity II HPLC (Agilent Technologies, Santa Clara, CA, USA) with a reserved-phase column (LiChrospher 100-5 RP18 250 mm × 4.0 mm, 5µm, Agilent Technologies), DAD (Agilent 1260 Infinity II DAD WR) and fluorescence detector (FLD) (Agilent 1260 Infinity II FLD Spectra) as previous publications [24,25]. The mobile phases were 50 mM ammonium dihydrogen phosphate pH 2.6 (mobile phase A), 20% (*v*/*v*) mobile phase A in acetonitrile (mobile phase B), 0.2 M *ortho*-phosphoric acid in the water, pH 1.5 (mobile phase C). The detailed gradient was as previously published [26]. The column temperature was maintained at 40 °C with a flow rate of 0.5 mL/min. An amount of 20 µL of sample supernatant was injected. The monomeric phenolics were identified and quantified at different wavelengths: 280 nm for gallic acid, 316 nm for hydroxycinnamic acids, 360 nm for flavonols, and 520 nm for anthocyanins. (+)-catechin and (-)-epicatechin were detected and quantified using FLD with an excitation wavelength at 276 nm and emission wavelength at 316 nm. Flavan-3-ols were quantified using (-)-epicatechin as the reference standard. Hydroxycinnamic acids were quantified using caffeic acid as the reference standard. Flavonols were quantified using quercetin-3-*O*-glucoside as standard. Anthocyanins were quantified using malvidin-3-*O*-glucoside (M3G) and malvidin-3,5-*O*-diglucoside (M35DG) as standards for mono- and di-glucoside, respectively. The information of calibration curves and related parameters (regression equation, coefficient of correlation, etc.) were listed in Table S2 (Supplementary data). Analyses were conducted in duplicate for each sample.

### 4.6. Total Iron-Reactive Phenolic Compounds and Condensed Tannin Analysis

Total iron-reactive phenolic compounds and condensed tannins in must and wine samples were quantified using the “Adams–Harbertson” bovine serum albumin (BSA) precipitation assay [27,28]. Briefly, the must or wine samples were incubated with a buffer containing 5% triethanolamine (*v*/*v*) and 5% sodium dodecyl sulfate (*w*/*v*) where after the total iron-reactive phenolic compounds were determined based on the absorbance values at 510 nm before and after the reaction with ferric chloride. Tannins were quantified after precipitation with BSA and reaction with ferric chloride. The absorbance values were measured at 510 nm using semi-micro 1.5 mL cuvettes (Fisher Scientific, Pittsburgh, PA, USA) with a UV-Visible spectrophotometer (Genesys 150, ThermoScientific, Waltham, MA, USA). Total iron-reactive phenolics and tannin contents were quantified using (+)-catechin as an equivalent (CE). Analyses were conducted in duplicate for each sample.

### 4.7. Statistical Analysis

Statistical analyses were performed by one-way analysis of variance (ANOVA) with the post hoc Tukey’s HSD significant difference test (α = 0.05). All the statistical analyses were performed with the JMP^®^ Pro 16.1.0 software (SAS, Cary, NC, USA).

## 5. Conclusions

The effect of applying ACE treatment at crushing (ACE-C) and 24 h before pressing (ACE-P) on pH, TA, alcohol concentration, color characteristics (hue and color intensity), phenolic compounds concentration of Marquette wines were investigated throughout the winemaking process. ACE-C treatment exhibited a positive impact on the extraction of monomeric phenolic compounds and enhancement of red wine color during aging. These results indicate that the ACE application time affects the efficiency of the extraction of phenolic compounds. As a mechanical process to break down skin tissue into smaller fragments, ACE effectively facilitated the release of phenolic compounds from disrupted skin cells at crushing and provided an intensified red color in bottled wines, stable over five months of aging. Based on the results of instrumental color measurement, ACE could be considered by the wine industry to produce long-term color-stable red wine made from cold-hardy grapes. However, the condensed tannins content in Marquette wines was below the limit of quantification of protein precipitation method for all the treatments and, therefore, ACE treatment did not improve it, which was inconsistent with ACE-treated *V. vinifera* wine. Future work will be focusing on understanding the binding affinity of tannins with other macromolecules in interspecific hybrid grapes.

## Figures and Tables

**Table 1:** Chemical characteristics of the Marquette grape musts and wines at crushing, bottling, and after 5 months aging. Values are mean ± standard deviation (*n* = 2).

Time Point	Treatment	°Brix	pH	TA ^1^ (g/L)	Ethanol (vol %)	Tartaric Acid (g/L)	Malic Acid (g/L)	Lactic Acid (g/L)
Crushing	ACE-C	25.75 ± 0.07 A ^2^	3.32 ± 0.02 Ab ^3^	13.31 ± 0.53 Aa	-	4.94 ± 0.43 Aa	10.06 ± 0.11 A	-
	ACE-P	24.85 ± 0.64 A	3.33 ± 0.01 Ab	12.09 ± 1.19 Aa	-	4.72 ± 0.23 Aa	10.04 ± 0.31 A	-
	CTL	25.35 ± 0.07 A	3.31 ± 0.01 Ac	11.53 ± 1.46 Aa	-	4.59 ± 0.02 Aa	10.40 ± 0.16 A	-
Bottling ^4^	ACE-C	-	3.81 ± 0.01 Ca	8.25	14.75	3.67	nd ^5^	3.37
	ACE-P	-	3.91 ± 0.02 Aa	7.50	13.64	2.89	nd	3.56
	CTL	-	3.90 ± 0.01 Ba	7.69	13.75	2.95	nd	3.35
Aging	ACE-C	-	3.79 ± 0.01 Ba	8.23 ± 0.12 Ab	14.57 ± 0.01 A	3.61 ± 0.10 Ab	nd	3.32 ± 0.02 C
	ACE-P	-	3.88 ± 0.01 Aa	7.29 ± 0.31 Bb	13.52 ± 0.03 C	3.06 ± 0.10 Cb	nd	3.35 ± 0.01 B
	CTL	-	3.87 ± 0.01 Ab	7.57 ± 0.26 Bb	13.63 ± 0.03 B	3.27 ± 0.04 Bb	nd	3.40 ± 0.01 A

^1^ Titratable acidity, tartaric acid equivalent. ^2^ Different uppercase letters within the same column indicate significant differences in the content of the measured variables among the different treatments (*p* < 0.05) within same time point. ^3^ Different lowercase letters within the same column indicate significant differences in the content of the measured variables among the different time points (*p* < 0.05) within same sample. ^4^ The chemical properties of sample at bottling only measured once, except for pH. ^5^ nd, not detected.

**Table 2:** Monomeric anthocyanins content (mg/L) in Marquette grape musts and wines at crushing, bottling, and after 5 months aging. Values are mean ± standard deviation (*n* = 2).

Time Point	Treatment	Unknown 1	Delphinidin-3-*O*-glucoside	Peonidin-3,5-*O*-diglucoside	Malvidin-3,5-*O*-diglucoside	Unknown 2	Malvidin-3-*O*-glucoside	Total Anthocyanins
Crushing	ACE-C	nd ^3^	nd	22.30 ± 5.61 A ^1^ a ^2^	92.32 ± 28.56 Ab	nd	30.52 ± 16.04 Ab	145.14 ± 50.22 Ac
	ACE-P	nd	nd	16.03 ± 0.96 Ab	61.28 ± 6.90 Ac	nd	13.83 ± 4.61 Ac	91.14 ± 12.47 Ac
	CTL	nd	nd	14.95 ± 1.50 Ab	54.49 ± 3.64 Ab	nd	nq ^4^	79.32 ± 6.94 Ac
Bottling	ACE-C	38.69 ± 4.74 Aa	25.11 ± 2.80 Aa	29.51 ± 6.54 Aa	175.27 ± 22.05 Aa	34.04 ± 1.88 Aa	56.65 ± 3.45 Aa	359.27 ± 41.41 Aa
	ACE-P	36.76 ± 3.33 Aa	18.47 ± 1.95 B	28.55 ± 5.52 Aa	165.93 ± 18.87 Aa	24.78 ± 1.26 Ba	44.83 ± 1.99 Ba	319.31 ± 32.50 Aa
	CTL	40.16 ± 3.63 Aa	19.04 ± 1.42 B	30.09 ± 4.82 Aa	178.54 ± 22.86 Aa	27.64 ± 1.43 Ba	53.74 ± 2.98 Aa	349.22 ± 37.09 Aa
Aging	ACE-C	30.32 ± 0.30 Bb	17.20 ± 0.27 b	23.47 ± 0.61 Ba	140.93 ± 1.17 Ba	24.77 ± 1.18 Ab	39.95 ± 0.64 Ab	276.65 ± 3.79 Ab
	ACE-P	29.13 ± 0.49 Cb	nq	23.67 ± 1.56 Bab	139.24 ± 1.59 Bb	17.10 ± 0.19 Cb	31.84 ± 0.67 Bb	240.97 ± 3.33 Bb
	CTL	32.51 ± 0.40 Ab	nq	26.28 ± 1.46 Aa	155.30 ± 2.70 Aa	19.34 ± 0.82 Bb	39.18 ± 0.91 Ab	272.63 ± 5.57 Ab

^1^ Different uppercase letters within the same column indicate significant differences in the content of the measured variables among the different treatments (*p* < 0.05) within same time point. ^2^ Different lowercase letters within the same column indicate significant differences in the content of the measured variables among the different time points (*p* < 0.05) within same sample. ^3^ nd, not detected. Values below detection limit. ^4^ nq, not quantified. Values below quantification limit.

**Table 3:** Monomeric non-anthocyanin phenolic compounds content (mg/L) in Marquette grape musts and wines at crushing, bottling, and after 5 months aging. Values are mean ± standard deviation (*n* = 2).

Time Point	Treatment	Gallic Acid	(+)-Catechin	(-)-Epicatechin	Caftaric Acid	Quercetin-3-*O*-glucoside	Myricetin	Quercetin	Total Phenolics ^1^
Crushing	ACE-C	1.66 ± 2.35 c ^2^	20.44 ± 11.25 A ^3^b	8.05 ± 3.94 Ab	10.39 ± 2.46 Ab	2.79 ± 0.99 Ab	nd ^4^	nd	43.34 ± 20.99 Ab
	ACE-P	nd	14.56 ± 8.96 Ab	5.42 ± 4.26 Ab	5.88 ± 2.40 Ab	2.45 ± 0.37 Ab	nd	nd	28.31 ± 15.99 Ab
	CTL	nd	7.42 ± 2.33 Ab	2.44 ± 1.04 Ab	4.96 ± 2.91 Ab	1.95 ± 0.40 Ac	nd	nd	16.77 ± 5.88 Ab
Bottling	ACE-C	103.29 ± 0.49 Ab	90.70 ± 0.96 Aa	36.76 ± 0.51 Aa	27.44 ± 0.03 Aa	5.62 ± 0.09 Aa	2.78 ± 0.00 Ab	nq ^5^	266.59 ± 1.04 Aa
	ACE-P	80.10 ± 0.20 Bb	64.81 ± 1.01 Ba	27.15 ± 0.14 Ba	20.56 ± 0.17 Ba	3.71 ± 0.04 Ca	2.01 ± 0.02 Cb	nq	198.35 ± 1.20 Ba
	CTL	77.34 ± 0.45 Cb	58.11 ± 0.20 Ca	24.07 ± 0.19 Ca	20.44 ± 0.00 Ba	4.29 ± 0.06 Ba	2.35 ± 0.00 Bb	nq	186.61 ± 0.00 Ca
Aging	ACE-C	113.63 ± 1.95 Aa	84.74 ± 1.16 Aa	35.29 ± 0.36 Aa	27.16 ± 0.03 Aa	4.38 ± 0.02 Aa	3.71 ± 0.03 Aa	nq—1.79	269.73 ± 1.15 Aa
	ACE-P	86.71 ± 0.83 Ba	61.60 ± 0.56 Ba	26.83 ± 0.38 Ba	20.63 ± 0.15 Ba	2.69 ± 0.12 Cb	2.44 ± 0.02 Ca	nq—1.56	201.30 ± 2.54 Ba
	CTL	83.29 ± 1.27 Ca	57.85 ± 0.80 Ca	24.93 ± 0.45 Ca	20.05 ± 0.06 Ca	3.23 ± 0.14 Bb	3.05 ± 0.03 Ba	nq—2.17	193.45 ± 1.81 Ca

^1^ Sum of all monomeric non-anthocyanin phenolic compounds as listed in the table. ^2^ Different lowercase letters within the same column indicate significant differences in the content of the measured variables among the different time points (*p* < 0.05) within same sample. ^3^ Different uppercase letters within the same column indicate significant differences in the content of the measured variables among the different treatments (*p* < 0.05) within same time point. ^4^ nd, not detected. Values below detection limit. ^5^ nq, not quantified. Values below quantification limit.

## Supplementary Materials

The following are available online, Table S1: Compounds identification, retention time, regression equation, coefficient of correlation (R2), limit of detection, and limit of quantification by HPLC-DAD and RID, Table S2: Reference standard compounds of monomeric phenolics, retention time, regression equation, coefficient of correlation (R2), limit of detection, and limit of quantification by HPLC DAD or FLD.

## References

[1] De Freitas V.A.P., Fernandes A., Oliveira J., Teixeira N., Mateus N. (2017). A review of the current knowledge of red wine colour. OENO One.

[2] Kennedy J.A., Saucier C., Glories Y. (2006). Grape and Wine Phenolics: History and Perspective. Am. J. Enol. Vitic..

[3] Lingua M.S., Fabani M., Wunderlin D.A., Baroni M.V. (2016). From grape to wine: Changes in phenolic composition and its influence on antioxidant activity. Food Chem..

[4] Burtch C.E., Mansfield A.K., Manns D.C. (2017). Reaction Kinetics of Monomeric Anthocyanin Conversion to Polymeric Pigments and Their Significance to Color in Interspecific Hybrid Wines. J. Agric. Food Chem..

[5] Rivas-Gonzalo J.-C., Bravo-Haro S., Santos-Buelga C. (1995). Detection of Compounds Formed through the Reaction of Malvidin 3-Monoglucoside and Catechin in the Presence of Acetaldehyde. J. Agric. Food Chem..

[6] Busse-Valverde N., Gómez-Plaza E., López-Roca J.M., Gil-Muñoz R., Bautista-Ortín A.B. (2011). The Extraction of Anthocyanins and Proanthocyanidins from Grapes to Wine during Fermentative Maceration Is Affected by the Enological Technique. J. Agric. Food Chem..

[7] Kassara S., Kennedy J.A. (2011). Relationship between Red Wine Grade and Phenolics. 2. Tannin Composition and Size. J. Agric. Food Chem..

[8] Pedneault K., Provost C. (2016). Fungus resistant grape varieties as a suitable alternative for organic wine production: Benefits, limits, and challenges. Sci. Hortic..

[9] Manns D.C., Lenerz C.T.M.C., Mansfield A.K. (2013). Impact of Processing Parameters on the Phenolic Profile of Wines Produced from Hybrid Red Grapes Maréchal Foch, Corot noir, and Marquette. J. Food Sci..

[10] Springer L.F., Sacks G.L. (2014). Protein-Precipitable Tannin in Wines from Vitis vinifera and Interspecific Hybrid Grapes (Vitis ssp.): Differences in Concentration, Extractability, and Cell Wall Binding. J. Agric. Food Chem..

[11] Sparrow A.M., Holt H.E., Pearson W., Dambergs R.G., Close D.C. (2016). Accentuated Cut Edges (ACE): Effects of Skin Fragmentation on the Composition and Sensory Attributes of Pinot noir Wines. Am. J. Enol. Vitic..

[12] Sparrow A.M., Smart R.E., Dambergs R.G., Close D.C. (2015). Skin Particle Size Affects the Phenolic Attributes of Pinot noir Wine: Proof of Concept. Am. J. Enol. Vitic..

[13] Kang W., Bindon K.A., Wang X., Muhlack R.A., Smith P.A., Niimi J., Bastian S.E.P. (2020). Chemical and Sensory Impacts of Accentuated Cut Edges (ACE) Grape Must Polyphenol Extraction Technique on Shiraz Wines. Foods.

[14] Casassa L.F., Beaver C.W., Mireles M., Larsen R.C., Hopfer H., Heymann H., Harbertson J.F. (2013). Influence of Fruit Maturity, Maceration Length, and Ethanol Amount on Chemical and Sensory Properties of Merlot Wines. Am. J. Enol. Vitic..

[15] Jensen J.S., Werge H.H.M., Egebo M., Meyer A.S. (2008). Effect of Wine Dilution on the Reliability of Tannin Analysis by Protein Precipitation. Am. J. Enol. Vitic..

[16] He F., Liang N.-N., Mu L., Pan Q.-H., Wang J., Reeves M.J., Duan C.-Q. (2012). Anthocyanins and Their Variation in Red Wines I. Monomeric Anthocyanins and Their Color Expression. Molecules.

[17] González-Neves G., Gi lG., Barreiro L. (2008). Influence of grape variety on the extraction of anthocyanins during the fermentation on skins. Eur. Food Res. Technol..

[18] Morata A., Gómez-Cordovés M.C., Suberviola J., Bartolomé B., Colomo A.B., Suárez J.A. (2003). Adsorption of Anthocyanins by Yeast Cell Walls during the Fermentation of Red Wines. J. Agric. Food Chem..

[19] Moreno-Arribas M., Gómez-Cordovés C., Martín-Álvarez P. (2008). Evolution of red wine anthocyanins during malolactic fermentation, postfermentative treatments and ageing with lees. Food Chem..

[20] Watrelot A.A., Le Bourvellec C., Imberty A., Renard C.M. (2013). Interactions between Pectic Compounds and Procyanidins are Influenced by Methylation Degree and Chain Length. Biomacromolecules.

[21] Springer L.F., Sherwood R.W., Sacks G.L. (2016). Pathogenesis-Related Proteins Limit the Retention of Condensed Tannin Additions to Red Wines. J. Agric. Food Chem..

[22] Bindon K.A., Li S., Kassara S., Smith P.A. (2016). Retention of Proanthocyanidin in Wine-like Solution Is Conferred by a Dynamic Interaction between Soluble and Insoluble Grape Cell Wall Components. J. Agric. Food Chem..

[23] Nicolle P., Marcotte C., Angers P., Pedneault K. (2019). Pomace limits tannin retention in Frontenac wines. Food Chem..

[24] Gómez-Alonso S., García-Romero E., Hermosín-Gutiérrez I. (2007). HPLC analysis of diverse grape and wine phenolics using direct injection and multidetection by DAD and fluorescence. J. Food Compos. Anal..

[25] Ritchey J.G., Waterhouse A.L. (1999). A Standard Red Wine: Monomeric Phenolic Analysis of Commercial Cabernet Sauvignon Wines. Am. J. Enol. Vitic..

[26] Watrelot A.A., Badet-Murat M.-L., Waterhouse A.L. (2018). Oak barrel tannin and toasting temperature: Effects on red wine condensed tannin chemistry. LWT.

[27] Harbertson J.F., Picciotto E.A., Adams D.O. (2003). Measurement of Polymeric Pigments in Grape Berry Extracts and Wines Using a Protein Precipitation Assay Combined with Bisulfite Bleaching. Am. J. Enol. Vitic..

[28] Heredia T.M., Adams D.O., Fields K.C., Held P.G., Harbertson J.F. (2006). Evaluation of a Comprehensive Red Wine Phenolics Assay Using a Microplate Reader. Am. J. Enol. Vitic..

