# Supplementary material for: Effect of the Application Time of Accentuated Cut Edges (ACE) on Marquette Wine Phenolic Compounds
Source: Molecules. 2022 Jan 15;27(2):542. doi: 10.3390/molecules27020542 (PMC8779630; doi:10.3390/molecules27020542)
Supplement: Supplementary file 1 [file molecules-27-00542-s001.zip › molecules-1520745-SI.pdf]

Supplementary Materials.

Table S1. Compounds identification, retention time, regression equation, coefficient of correlation (R^2), limit of detection, and limit of quantification by HPLC-DAD and RID.

Compound	Regression Equation	R^2	Calibration Range (g/L)	Limit of Detection (g/L)	Limit of Quantification (g/L)
Ethanol	y = 53535x + 9534.3	0.9996	6.25-100	1.86	5.65
Tartaric acid	y = 115826x - 4322.9	1.0000	0.625-10	0.06	0.18
Malic acid	y = 736.1786x - 76.3664	0.9998	0.625-10	0.14	0.43
Lactic acid	y = 102915x + 4702.2	0.9990	0.3125-5	0.15	0.46

Table S2. Reference standard compounds of monomeric phenolics, retention time, regression equation, coefficient of correlation (R^2), limit of detection, and limit of quantification by HPLC DAD or FLD.

Reference Standard Compound	Regression Equation	R^2	Calibration Range (g/L)	Limit of Detection (g/L)	Limit of Quantification (g/L)
Malvidin-3-*O*-glucoside	y = 131.95x - 27.145	0.9993	1-200	4.19	12.71
Malvidin-3,5-*O*-diglucoside	y = 105.69x + 7.9169	0.9991	1-200	4.71	14.29
(-)-Epicatechin (DAD)	y = 40.566x - 2.0108	1.0000	1-100	0.33	1.01
(-)-Epicatechin (FLD)	y = 12.871x - 2.1446	0.9999	0.1-100	0.70	2.11
Caffeic acid	y = 313.52x - 40.484	1.0000	1-100	0.36	1.10
Quercetin-3-*O*-glucoside	y = 113.01x - 10.082	1.0000	1-100	0.48	1.46
